# A Routing-Based Repair Method for Digital Microfluidic Biochips Based on an Improved Dijkstra and Improved Particle Swarm Optimization Algorithm

**DOI:** 10.3390/mi11121052

**Published:** 2020-11-28

**Authors:** Wenbin Zheng, Jinlong Shi, Anqi Wang, Ping Fu, Hongyuan Jiang

**Affiliations:** 1 School of Electronics and Information Engineering, Harbin Institute of Technology, Harbin 150001, China; zhengwenbin@hit.edu.cn (W.Z.); shijinlong@hit.edu.cn (J.S.); wanganqi940504@163.com (A.W.); 2 School of Mechatronics Engineering, Harbin Institute of Technology, Harbin 150001, China; jhy_hit@sina.com

**Keywords:** DMFB, fault repair, routing-based, improved Dijkstra algorithm, improved particle swarm optimization algorithm

## Abstract

Digital microfluidic biochips (DMFBs) are attractive instruments for obtaining modern molecular biology and chemical measurements. Due to the increasingly complex measurements carried out on a DMFB, such chips are more prone to failure. To compensate for the shortcomings of the module-based DMFB, this paper proposes a routing-based fault repair method. The routing-based synthesis methodology ensures a much higher chip utilization factor by removing the virtual modules on the chip, as well as removing the extra electrodes needed as guard cells. In this paper, the routing problem is identified as a dynamic path-planning problem and mixed path design problem under certain constraints, and an improved Dijkstra and improved particle swarm optimization (ID-IPSO) algorithm is proposed. By introducing a cost function into the Dijkstra algorithm, the path-planning problem under dynamic obstacles is solved, and the problem of mixed path design is solved by redefining the position and velocity vectors of the particle swarm optimization. The ID-IPSO routing-based fault repair method is applied to a multibody fluid detection experiment. The proposed design method has a stronger optimization ability than the greedy algorithm. The algorithm is applied to 8×9, 8×8, and 7×8 fault-free chips. The proposed ID-IPSO routing-based chip design method saves 13.9%, 14.3%, and 14.5% of the experiment completion time compared with the greedy algorithm. Compared with a modular fault repair method based on the genetic algorithm, the ID-IPSO routing-based fault repair method has greater advantages and can save 39.3% of the completion time on average in the completion of complex experiments. When the ratio of faulty electrodes is less than 12% and 23%, the modular and ID-IPSO routing-based fault repair methods, respectively, can guarantee a 100% failure repair rate. The utilization rate of the electrodes is 18% higher than that of the modular method, and the average electrode usage time is 17%. Therefore, the ID-IPSO routing-based fault repair method can accommodate more faulty electrodes for the same fault repair rate; the experiment completion time is shorter, the average number of electrodes is lower, and the security performance is better.

## 1. Introduction

With the continuing revolution in fabrication and packaging processes, microfluidics-based biochips, also referred to as labs on chips, have the potential to replace conventional laboratory equipment due to their limited need for human intervention, their portability, and their high throughput and sensitivity [1,2]. These instruments can be applied in various areas, such as drug discovery, clinical diagnosis, DNA sequencing, protein analysis, and immunoassays [3,4]. Clinical diagnosis is one of the important links in disease diagnosis. Traditional clinical disease detection usually relies on the large testing equipment of a testing center, which not only takes a long time to detect but also consumes a large amount of reagents and faces great controversy due to the need to collect many biological specimens such as blood [5,6,7]. Digital microfluidic biochips (DMFBs) use discrete amounts of fluids of nanoliter volume, named droplets, to perform operations such as dispensing, transport, mixing, splitting, and detection. Many chemical and biological measurements can benefit from the small size of these biochips; bioassay protocols are scaled down in terms of both sample volumes and completion times, and they can be performed in a significantly smaller laboratory space. At present, the applications of digital microfluidic chips mainly focus on the biological and medical fields, where all kinds of body fluids can be moved and analyzed [8,9,10]. DMFBs are widely used in clinical diagnostics and require high-precision output for each operation.

**Figure 1:**
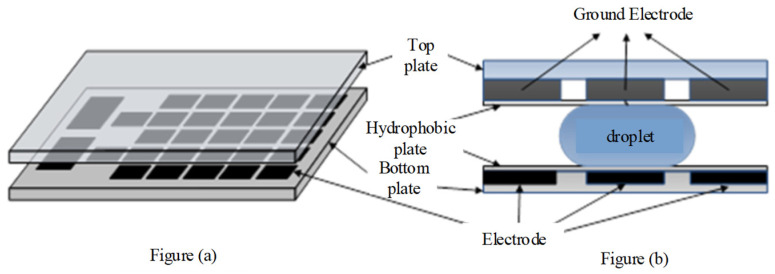
(**a**) Two-dimensional (2D) electrode array. (**b**) Cross-sectional view of the digital microfluidic biochip (DMFB).

On the DMFB analysis system, each droplet can be controlled independently, and each cell in the array has the same structure. Therefore, a DMFB consists of a two-dimensional (2D) electrode array and some peripheral devices, including the detecting sensor and dispensing port, as shown in Figure 1. Figure 1a shows a 2D electrode array. Figure 1b shows a cross-sectional view of the DMFB. The basic droplet movement is based on the electrowetting on dielectric (EWOD) principle [11,12,13]. Hence, the operations can be executed anywhere on the chip by occupying a set of electrodes in a reconfigurable way. By applying a series of voltages to adjacent control electrodes, the droplets between the top and bottom plates move to different cells, as expected. The sensors on the DMFB have multiple functions. The reaction product can be detected by an integrated light-emitting diode (LED)–photodiode sensor. A DMFB with a sensing feedback signal can monitor intermediate products during execution [14].

The design methods of DMFBs are mainly divided into two types: modularization design [15] and droplet path design [16]. The former is widely used and more popular, and its core idea is to define an electrode area on the chip as the corresponding module. When the biochemical experiment needs to complete this operation, it only needs to send the droplet into the module. The authors of [17] presented the first module and sensor co-placement algorithm for cyber-physical digital microfluidic biochips that considered the sensor constraint and minimized the actuation times of the electrodes. The authors of [18] first formalized the error-recovery objectives and then synthesized the optimal error-recovery protocols using a model based on stochastic multiplayer games (SMGs). These works also presented a global error-recovery technique that can update the schedule of fluidic operations in an adaptive manner. Using three representative real-life bioassays, these works showed that the proposed approach can effectively reduce the bioassay completion time and increase the probability of success for error recovery. The authors of [19] first proposed an online synthesis strategy that resynthesizes the application at runtime when operations experience variability in their execution time, thus exploiting the slack to obtain shorter application completion times. These works also proposed a quasi-static synthesis strategy that determines a database of alternative implementations offline. During the execution of an application, several implementations are selected on the basis of the current execution scenario with operation execution time variability. The proposed strategies were evaluated using several benchmarks and compared to related works.

This modularization design idea has a simple design process and good portability [20]; however, after defining the electrode area as a functional module, it cannot fully take advantage of the reconfigurability of a digital microfluidic chip, and there are problems such as resource waste in the design process [21]. The design idea of a digital microfluidic chip based on a droplet path plans the droplet’s movement path directly. Different biochemical experiments need to be redesigned, but personalized chip design can be carried out according to specific requirements. For droplet path design approaches, a series of solutions have been proposed. The authors of [22] proposed a fluid-level design for DMFBs that considered cross-contamination and degrading electrodes together, and a graph model was used to address them. An exact algorithm was presented. The obtained results were validated with several benchmarks. A comprehensive routing solution was also proposed for DMFB chip-level designs in [23]. This paper presented an exact routing method to find the minimal solution with respect to the completion time. For the first time, this allowed the benefits of DMFBs to be evaluated in comparison to their conventional counterparts, as well as allowing a quality assessment of previously proposed routing methods in this domain to be conducted. The authors of [24,25,26] proposed the module-less synthesis (MLS) process and modified MLS process to ensure improved security measures and demonstrated various attack scenarios for the modified MLS method on a DMFB. These works also proposed a checkpoint-based novel attack detection method for the modified MLS technique. The authors of [27] proposed a novel heuristic routing technique for DMFB architecture to address routing complexities due to overlapping nets, interfering blockages, and deadlock zones formed by conflicting nets. These works categorized various region-based movements of droplets on a chip and derived a metric named the snooping index to improve the routing performance of the droplets in the first phase. Next, an exhaustive search was applied to find the routing path for the remaining nets considering different constraints specific to the DMFB platform.

Routing-based fault repair method design removes the concept of a resource module, allowing the operation to perform biochemical experiments via any electrode sequence on the array. We call this approach the routing-based synthesis method [28,29,30]. In the design process, the main operation that needs to be designed is the reconfigurable operation, namely, the mixing operation. The mixing operation needs to complete two steps. First, the electrodes are controlled to make the two droplets that are to be mixed move to the same position, and then the droplets can move along any path to complete the mixing operation. Therefore, this task requires designing the droplet path of the mixing operation in order to complete the biochemical experiment. The mixing operation needs to complete two mixed droplet movements in the same location and find the shortest path between the two droplets. The chip of failure electrodes and other droplets can be regarded as failing, and then the problem can be considered equivalent to the path-planning problem; in an environment with obstacles, a collision-free path from start to finish is sought. In the first step, the shortest path between the two droplets is to be found. This problem is Non-deterministic Polynomial (NP ) hard [31]. The main feature of the traditional Dijkstra algorithm [32,33,34] is to search for a path from the starting point to all nodes on the outside until all nodes in the map are found. That is, the shortest path from start to finish is found by breadth-first traversing all nodes in the map. Because this algorithm traverses all nodes to obtain the shortest path, it can ensure the global optimal solution; however, there are many traversal nodes with low efficiency, and this strategy is not suitable for the obstacle dynamics of this task. In the second step, the droplet movement path is designed to complete the mixing operation. The particle swarm optimization (PSO) algorithm is a kind of evolutionary algorithm that starts from a random solution, finds the optimal solution through iteration, and evaluates excellent solutions by using a fitness function [35,36,37]. Its evolution rules are simple, and the global optimal solution is determined by following the global and local optimal values currently found. Because of its high parallelism, the PSO algorithm is easy to implement and converges quickly; thus, it is suitable for solving large-scale path-planning problems. The authors of [21] eliminated the concept of virtual devices and allowed the droplets to move on the chip along any route during operation execution. Thus, the synthesis problem was transformed into a routing problem. An algorithm was developed on the basis of a greedy randomized adaptive search procedure (GRASP); we show that routing-based synthesis leads to significant improvements in the application completion time compared to traditional synthesis based on virtual devices. However, the routing of droplets becomes more complex and difficult when the electrodes on the chip fail. Therefore, the disadvantage of the routing-based approach is that the fault tolerance of the chip is very weak and, hence, the fault tolerance of the chip needs to be considered in the design of the chip.

This paper proposes an improved Dijkstra and improved particle swarm optimization (ID-IPSO) routing-based fault repair method. The routing problem is identified as a dynamic path-planning problem and mixed path design problem under certain constraints. By introducing a cost function into the Dijkstra algorithm, the path-planning problem under dynamic obstacles is solved, and the problem of mixed path design is solved by redefining the position and velocity vectors of particle swarm optimization. The ID-IPSO routing-based fault repair method is applied to a multibody fluid detection experiment. Through the combination of the ID and IPSO algorithms, a DMFB routing-based fault repair method is proposed; a humoral detection experiment is taken as the verification experiment, and various experiments with different humoral detections are applied to the routing-based and modularized DMFB fault repair method.

The remainder of this paper is organized as follows: Section 2 presents a mathematical model of routing-based methods. Section 3 presents the ID-IPSO routing-based fault repair method, including path scheduling based on the ID algorithm and droplet mixing path design based on the IPSO algorithm. Section 4 and Section 5 show the experimental results and conclude this paper, respectively.

## 2. Mathematical Model of Routing-Based Methods

**Figure 2:**
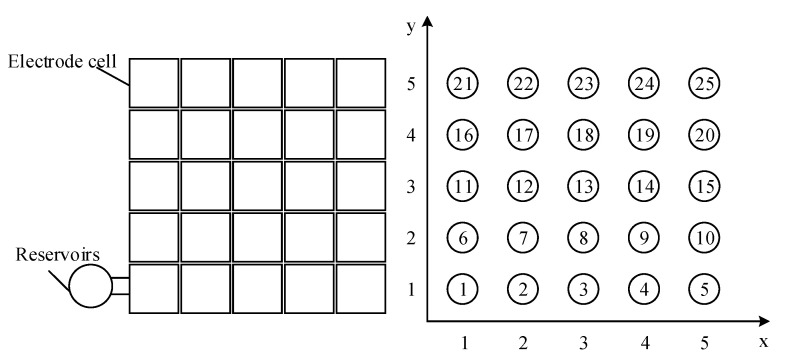
A microelectrode array distribution with 25 electrodes.

Routing-based repair of a digital microfluidic chip involves designing the chip under the condition of failure and ensuring that the biochemical experiment is completed on the broken chip in the minimum period of time. A digital microfluidic chip is an array of electrodes formed by a series of electrodes in a certain layout. In this study, only shape rules were considered, and the electrode array was a rectangular DMFB chip. A mathematical model of the DMFB electrode array was established, as shown in Figure 2. The mathematical model was composed of a 25-electrode array with five rows and five columns.

**Constraints:** Two droplets should not be placed directly adjacent to each other or aligned along a diagonal line; otherwise, the droplets will exhibit unanticipated fusion. The locations of the two droplets are $\left(m_{i},n_{i}\right)$ and $\left(m_{j},n_{j}\right)$, and the static fluid constraint condition can be expressed as

(1) $$\left\{ \begin{array}{l} \left|m_{i}-m_{j}\right|\geq 1\\ \left|n_{i}-n_{j}\right|\geq 1\\ \left|m_{i}-m_{j}\right|+\left|n_{i}-n_{j}\right|\geq 3 \end{array}\right..$$

**Objection:** DMFB fault repair reconstructs the chip design according to the needs of the biochemical experiment under the condition of failure. The goal is to ensure the shortest completion time of the experiment. Fault repair on the chip is constrained by the fault constraints and time constraints. The actual experiment completion time cannot exceed the maximum completion time required by the biochemical experiment. The maximum completion time is expressed as $T_{real}$, and the total number of operations is expressed as K. The completion time required for operation l is $T_{l}$; then, the experimental completion time is expressed as Equation (2).

(2) $$T_{real}=\sum_{l=1}^{K}T_{l}.$$

The objective is to minimize the completion time of the experiment, as well as to achieve the objective.

(3) $$T_{\min}=\min\left\{T_{real}\right\}.$$

## 3. The Improved Dijkstra and Improved Particle Swarm Optimization (ID-IPSO) Algorithm

Routing-based DMFB fault repair aims to complete the corresponding biochemical experiments on a chip with a fault using the routing-based repair method, and it involves the design and planning of the droplet path for the operation of the controlled droplets moving on the chip. In all biochemical operations, in addition to separating liquid droplets from each other and splitting the droplets, the moving path of the droplets needs to be designed. The hybrid operation needs to first be designed to move droplets in different electrode units to the same path by moving the shortest path to this path, which is called the path planning problem. Then, the droplets are controlled to move along a certain path. The completion percentage continuously accumulates during the movement process until the mixing or dilution ratio reaches 100%. This is called a hybrid path design problem.

In this paper, a grid method was used to build a map model for digital microfluidic chips. The ID algorithm was used to solve the problem of droplet path planning. The main idea of the traditional Dijkstra algorithm is to search all nodes with the starting point as the center to all the nodes in the map. That is, through the breadth-first traversal of all nodes in the map, the shortest path from the beginning to the end is obtained. The traditional algorithm traverses many nodes, has low efficiency, and does not apply to the dynamic situation of this task. In this paper, we propose an ID algorithm, a heuristic search factor, and a cost function. This algorithm can use the path selection heuristic and search the path in the direction of the most likely optimal solution. The problem of droplet mixing path design is solved by the IPSO algorithm. The IPSO algorithm begins with a random feasible solution and optimizes the objective function according to the evolutionary method. The problem of path design in this project cannot yield a feasible solution at random. Only in the process of continuous search can the optimal solution be obtained. In this paper, the IPSO algorithm was used to redefine the position vectors and velocity vectors of the particles, and the IPSO algorithm was used to optimize the droplet mixing path.

### 3.1. Path Scheduling Based on the ID Algorithm

The traditional Dijkstra algorithm can access each point only once, and according to the idea of the greedy algorithm, every search process ensures the shortest node in the current path. The cost function is introduced into the Dijkstra algorithm to improve the heuristic method. There are two kinds of fault points on the map: faulty electrodes and other droplets. The position of a faulty electrode does not change with time; however, the position of the other droplets varies with time, and the droplets can only move one electrode unit between adjacent time slices. The Dijkstra algorithm finds the shortest path in the current time slice according to the current fault mode because the fault location changes in the next time slice.

In the next location path calculation, we need to consider three areas: first, we consider selecting the shortest path to ensure the current path; second, we consider the nearest point to the terminal distance that does not have a fault; third, we select the moving position that is the farthest from the fault point. With the introduction of the cost function, the breadth-first search mechanism in all directions can be improved into a directional depth-first search mechanism. In the process of selection, the influences of a variety of factors are integrated to avoid obtaining a local optimal solution in the dynamic failure condition.

The cost function is used to estimate the cost of moving from a point to the endpoint. A greater value of the cost function results in a longer path-planning path. A path that continues to be searched may deviate from the optimal solution or fall into a local optimum. A smaller cost function results in a greater likelihood of the algorithm continuing to search in that direction. The cost function is defined as

(4) $$f_{\cos t}(D_{k,i})=a\times dis(D_{k,i},D_{k,i,start})+b\times dis(D_{k,i},D_{k,i,end})+\frac{g}{min\left[dis(D_{k,i},E^{f})\right]},$$

where $dis(D_{k,i},D_{k,i,start})$ represents the distance from the point to the beginning node, $dis(D_{k,i},D_{k,i,end})$ indicates the distance between the point and the endpoint, and $\min\left[dis(D_{k,i},E^{f})\right]$ indicates the distance between the point and the nearest fault point. The closer a point is to the starting node, the closer it is to the endpoint, and the farther it is from the fault point, the smaller the cost function is of the point and, thus, its selection priority is higher.

### 3.2. Droplet Mixing Path Design Based on IPSO

The PSO algorithm is based on the behavior of a group of organisms, such as a group of insects, birds, or fish. The PSO algorithm takes a population (such as birds or particles) to solve the target problem and uses them to test the search space. A particle $X_{i}={\left(x_{i1},x_{i2},\ldots ,x_{in}\right)}^{T}$ is defined as the position of a moving particle in an *N*-dimensional space. For each dimension, each bird in the population has an adjustable velocity (change in position) $V={\left(v_{i1},v_{i2},\ldots ,v_{in}\right)}^{T}$ depending on its position in the search space. The performance of each particle is evaluated by an objective function. The particles in the optimal position in the current population are specified as follows: the local best $X_{lb}$, the index of the best particle position of all previous iterations, and the global best $X_{gb}$ are stored and used for obtaining the new particle speed and position. The following equations describe the updates of the position and velocity:

(5) $$v_{id}{}^{n+1}=v_{id}{}^{n}+c_{1}r_{1}(X_{lb}{}^{n}-x_{id}{}^{n})+c_{2}r_{2}(X_{gb}{}^{n}-x_{id}{}^{n}),$$

(6) $$x_{id}{}^{n+1}=x_{id}{}^{n}+v_{id}{}^{n},$$

where n represents the number of iterations, id is the current dimension, and the parameters $c_{1}r_{1}$ and $c_{2}r_{2}$ are uniformly distributed random numbers, which can prevent the algorithm from obtaining a local optimal solution. Parameters $c_{1}$ and $c_{2}$ are scalars that control the individuals and population, which affects the study ratio of the particles. Parameters $r_{1}$ and $r_{2}$ range from 0 to 1 and have a uniform distribution of random numbers.

$x_{i}{}^{t}$ represents the sequence number of the particle at any time. $X_{i}{}^{T}=(x_{i}{}^{1},x_{i}{}^{2},\ldots ,x_{i}{}^{T})$ is the position vector of the particle, representing the moving path of the particle i designed for the droplet. $X_{gb}^{T}=(x_{gb}{}^{1},x_{gb}{}^{2},\ldots ,x_{gb}{}^{T})$ represents the global optimum, and $X_{lb}{}^{T}=(x_{lb}{}^{1},x_{lb}{}^{2},\ldots ,x_{lb}{}^{T})$ represents the location vector of the locally optimal particles. The velocity vector is defined as the direction of motion of the droplets, indicated as $V_{i}{}^{t}$. The droplets have at most five kinds of motion at every moment: steady, upward, downward, left, and right.

The velocity direction of the particles is determined by the optimal solution of the historical population $X_{gb}{}^{T}$, the optimal solution of the current population $X_{lb}{}^{T}$, and the best choice at present $v_{i}{}^{t}$. The velocity direction of the particles is expressed as

(7) $$\vec{V_{i}{}^{t}}=c_{i}\vec{v_{i}{}^{t}}⊕c_{2}(x_{gb}{}^{t}-x_{gb}{}^{t-1})⊕(x_{lb}{}^{t}-x_{lb}{}^{t-1}).$$

In the first part, $v_{i}{}^{t}$, ensures the current maximum mixing direction. The method is as follows: the current motion is the same as that of the last time slice.

The second part is the information about the global optimal solution for the current search, that is, the moving direction of the global optimal solution at time t; the third part is the moving direction of the optimal solution of the current population.

Parameters $c_{1}$ and $c_{2}$ determine the influence of the current speed, global optimum, and local optimal solution on the current particle velocity. When determining the speed at the current time, a random number $r_{7}$ is generated, and the direction of the particle velocity is determined according to the following conditions:

(8) $$\begin{array}{l} \vec{V_{i}{}^{t}}=\left\{ \begin{array}{l} \vec{v_{i}{}^{t}}\;0<r_{7}\leq \frac{c_{1}r_{1}}{c_{1}r_{1}+c_{2}r_{2}+c_{3}r_{3}}\\ x_{gb}{}^{t}-x_{gb}{}^{t-1}\;\frac{c_{1}r_{1}}{c_{1}r_{1}+c_{2}r_{2}+c_{3}r_{3}}<r_{7}\leq \frac{c_{1}r_{1}+c_{2}r_{2}}{c_{1}r_{1}+c_{2}r_{2}+c_{3}r_{3}}\\ x_{lb}{}^{t}-x_{lb}{}^{t-1}\;\frac{c_{1}r_{1}+c_{2}r_{2}}{c_{1}r_{1}+c_{2}r_{2}+c_{3}r_{3}}<r_{7}\leq 1 \end{array}\right.. \end{array}$$

The particle location update function can be expressed as

(9) $$X_{i}{}^{t+1}=X_{i}{}^{t}⊕V_{i}{}^{t}.$$

With the increment of the time slice, the position of each particle is constantly updated, and the mixing degree of the droplets increases. The movement of the particles from a certain electrode unit to the adjacent electrode unit leads to an increase in the degree of mixing, which is defined as the percentage of the particles i that go from moving to completion. This value is defined as

(10) $$m_{i}{}^{t}=\left\{ \begin{array}{l} mix_{perhold\;}\;x_{i}{}^{t}=x_{i}{}^{t+1}\\ mix_{per0\;}\;<\vec{V_{k}{}^{t-1}},\vec{V_{k}{}^{t}}>=90^{\circ }\\ mix_{per180}\;<\vec{V_{k}{}^{t-1}},\vec{V_{k}{}^{t}}>=180^{\circ }\\ mix_{per0}\;<\vec{V_{k}{}^{t-1}},\vec{V_{k}{}^{t}}>=0^{\circ } \end{array}\right.,$$

where $m_{i}{}^{t}$ is the percentage of the cumulative mixing degree completed in time slice t for particle i; the cumulative percentage of the mixture is updated continuously as the particle position is updated until the particle satisfies $m_{i}{}^{t}>1$, and then the mixture is completed. The position vector of the particle is a feasible solution to the problem; the droplet is moved in order of the electrode unit provided by the particle position vector.

## 4. Experimental Results

### 4.1. Multiple Body Fluid Detection Experiment

**Figure 3:**
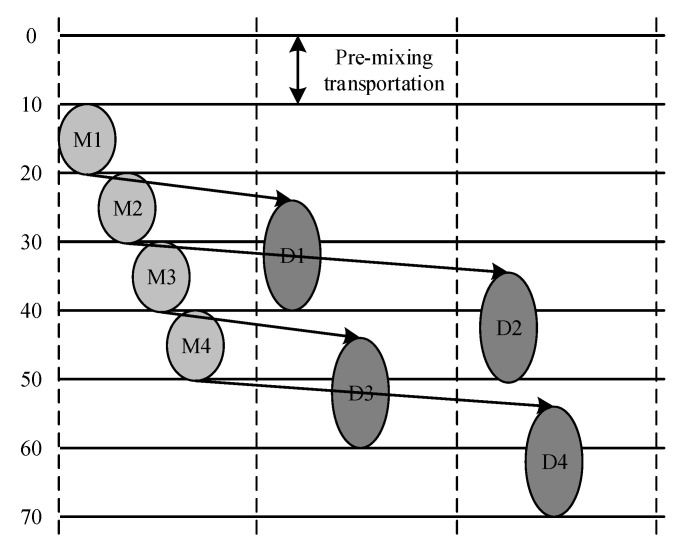
Schedule result for the multiplexed bioassay.

In this paper, the method of path design was used to perform an experiment with multiple body fluid detection. We mapped the multiplexed experiment to digital microfluidic biochips. The experiment with multiple body fluid detection was composed of a glucose assay and a lactate assay based on colorimetric enzymatic reactions. The mixing process of the experiment is shown in Figure 3. Two sample droplets and two reagent droplets were dispensed into the chip. They consisted of four pairs of droplets to be mixed, $\left\{S_{1},R_{1}\right\}$, $\left\{S_{1},R_{2}\right\}$, $\left\{S_{2},R_{1}\right\}$, and $\left\{S_{2},R_{2}\right\}$. Lastly, the analysis was completed by sequencing at the detection site.

### 4.2. Simulation of the Improved Dijkstra Algorithm

The routing-based fault repair method of a DMFB directly designs the droplet moving path, and the droplet on the chip should complete mixing, diluting, and transporting. The chip design method based on droplet routing needs to solve two problems: droplet transport and droplet mixing. Droplet transport moves the droplet from the starting point to the destination. Finding the shortest path from the starting point to the end is called path planning. There are two kinds of obstacles in this task: the first kind of obstacle is a faulty electrode, and the position of this obstacle does not change with time; the second kind of obstacle is the other droplets on the chip, and the obstacle locations change at all times. The digital microfluidic chip is reconfigurable; that is, electrodes can perform different operations in any position at any time. In one time slice, there can be other droplets on the chip. To verify the effectiveness of the ID algorithm for path planning, a fault point was set on a 10×10 chip, and the locations and movements of the other droplets were designed. As shown in the diagram, there were four other droplets on the chip. The experimental droplets should move from electrode $E_{1}(1,1)$ to position $E_{98}(9,10)$, and the parameters were α=0.2, β=0.4, and γ=0.7.

**Figure 4:**
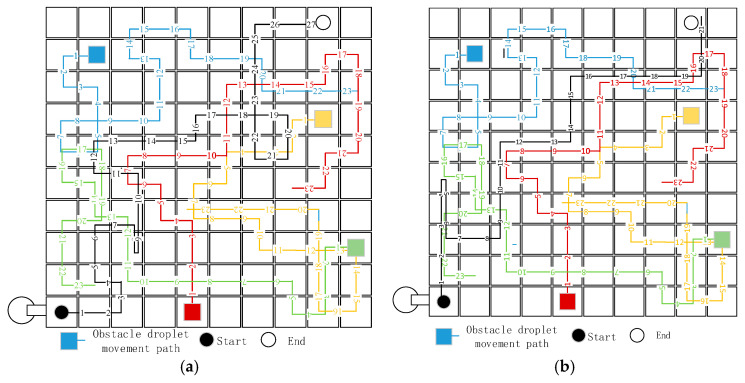
Path planning from the starting position to the end position in the traditional and improved Dijkstra algorithms. (**a**) The traditional Dijkstra algorithm (**b**) The improved Dijkstra algorithm.

The droplet paths designed by the traditional Dijkstra and ID algorithms are shown in Figure 4a,b, respectively. The two kinds of shortest droplet moving paths were 27 and 21, respectively, and the distance between the starting point and endpoint was decreased by the ID algorithm.

The traditional Dijkstra algorithm originates from the greedy algorithm: when searching a path, the shortest path among the current paths is always selected, and the whole problem is not considered. The Dijkstra algorithm gives priority to the shortest distance between the current path and the starting point and does not consider the distance between the current position and the endpoint. In the algorithm, when the droplet searches a path, the distance between the current droplet and the destination is chosen as the heuristic factor. Observing the paths of both can show that the ID algorithm is more systematic and objective in the path search process.

The path-planning problem based on digital microfluidic chip path planning is to design the shortest path under dynamic obstacles. The uncertainty of the locations of other droplets leads to a geometric rise in the solution space of the problem. At this time, the exhaustive method based on the depth-first traversal of the traditional Dijkstra algorithm does not have the advantage in solving the problem. For example, the path of the two-party planning in the graph shows that the Dijkstra algorithm would search the path without the guidance of the heuristic factor. The movement of the other droplets causes conflicts that increase the path length. In the ID algorithm proposed in this project, because the moving path of the other droplets is continuous, the fault location in the adjacent time slice has an association system, a fault heuristic factor is introduced in the Dijkstra algorithm, and a location far from the fault is chosen in the path. When the parameters are set, the parameters of the current path are affected. The factors affecting the distance between the current point and the end of the selection and the influencing factors of the distance between the current point and the obstacles when making choices focus on the location of other droplets and have a heuristic obstacle avoidance function.

### 4.3. Simulation of the Improved Particle Swarm Optimization (IPSO) Algorithm

The design of the droplet mixing path aims to optimize the path length under the constraint of a certain iteration termination condition under the premise of a known motion weight. In this paper, the IPSO algorithm was applied to 10×10 chips with one fault point to achieve five droplet mixing operations.

The number of particles per generation was 50, the total number of iterations was GEN = 200, and the parameters were set as $c_{1}=0.8$ and $c_{2}=0.3$.

**Figure 5:**
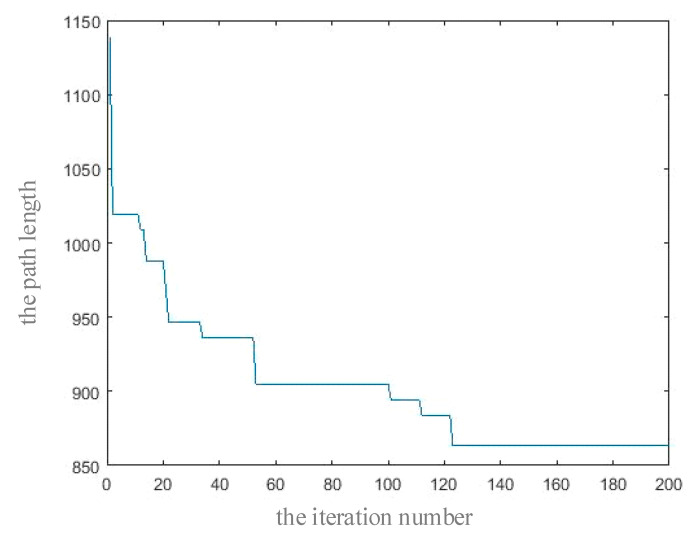
Convergence diagram of the improved Dijkstra algorithm.

To analyze the convergence of the algorithm, we used the iteration number as the horizontal axis and the optimal solution in each generation as the longitudinal axis to draw the convergence curve of the algorithm, as shown in Figure 5. As the iteration continued, the path length decreased continuously, and the path length became stable at 868 when the number of iterations was 122. The droplets needed 0.01 s for each moving electrode unit; thus, the droplets were mixed along the path, and the mixing time required was 8.86 s.

**Figure 6:**
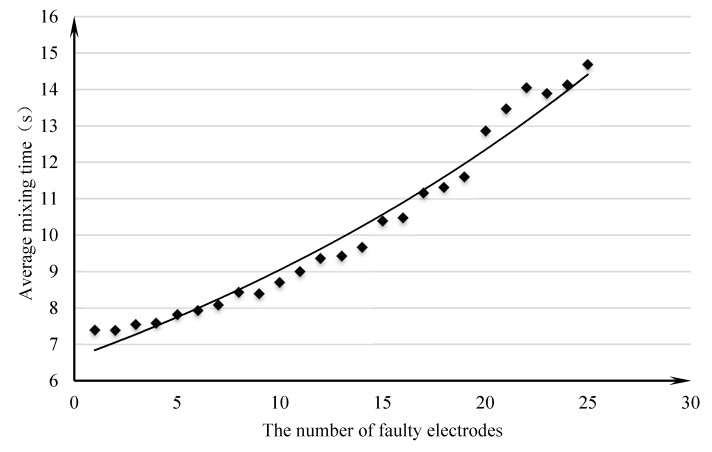
The relationship between the average mixing time and the number of faulty electrodes.

To verify the performance of the droplet mixing path design algorithm in the case of failure, the IPSO algorithm was applied to a chip with a breakdown number of 1–25 and an array size of 10×10, and five droplets and mixing paths were designed. The relationship between the average mixing time of five droplets and the number of faulty electrodes is shown in Figure 6.

The plot used the number of faulty electrodes as the abscissa and the mean mixing time as the ordinate to draw scatter plots and add trend lines. With the increase in the number of faulty electrodes, the mixing time increased gradually and fluctuated in some intervals. The randomness of the location of the faulty electrode and the uncertainty of the droplet moving path affected the completion time for some faulty electrodes. From the overall trend, the average mixing completion time increased exponentially. When there were more faulty electrodes, each additional faulty electrode increased the mixed completion time.

### 4.4. Simulation of the ID-IPSO Algorithm for a Routing-Based DMFB

To address the problems and defects of modular fault repair methods, this study designed a fault repair method using the idea of paths. Through the ID algorithm and the IPSO algorithm, the ID-IPSO routing-based fault repair method was implemented. The method proposed in this paper could also be designed on a chip without faults. The algorithm was applied to 8×9, 8×8, and 7×8 fault-free chips. The method of path design was used to complete an experiment with multiple body fluid detection. The proposed design method was compared with the greedy algorithm [21], and the experimental data are shown in Table 1.

The proposed ID-IPSO routing-based chip design method saved 13.9%, 14.3%, and 14.5% of the experiment completion time. The chip design method based on the greedy algorithm obtained the shortest moving direction of the current path when determining the moving path of the droplet. It was easy to obtain the optimal solution; only one search was carried out, and the optimization ability was not available. A heuristic factor was introduced into the search process to improve the path-planning ability. The IPSO algorithm was introduced, and swarm intelligence was used to search for the optimal solution.

To verify the performance of modular and routing-based fault repair methods, two fault repair methods were applied to multiple body fluid detection experiments. The performance parameters of the two methods were compared under the conditions of different scales of biochemical experiments and numbers of faulty electrodes. The advantages, disadvantages, and applicability of the two methods were analyzed.

First, the two methods were applied on 10×10 DMFB chips to complete multiple fluid tests with the patients. A total of 18 experiments were conducted, and, with the increase in the experimental sequence number, the test interval increased by 1 as the number of patients increased. Each patient underwent two fluid testing operations, and the fluid type and the test project were randomly determined. Over the 18 experiments, with the increase in the experimental sequence number, the experiment operand increased gradually. In the 18th experiment, 18 people were subjected to two tests with two body fluids, for a total of 288 operations.

**Figure 7:**
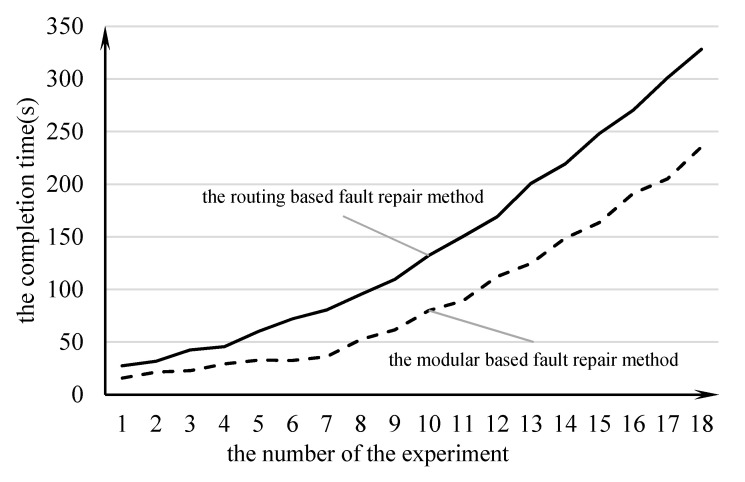
The relationship between the number of experiments and the completion time.

Figure 7 shows the number of the experiments and the completion time of the experiments. The figure shows that the complexity of an experiment increased as the number of patients increased. Under the same experimental conditions, the experimental completion time of the routing-based fault repair method was lower than the modular-based experiment completion time, and, with the increase in the number of patients, the difference between the two became increasingly large. In the completion of the more complex experiments, the path design method had a greater advantage and could save an average of 39.3% of the completion time.

To verify the fault repair performance based on modular fault repair and routing-based fault repair for different numbers of faults, a number of different fault points were set on a scale of 10×10 DMFB chips, and the number of fault points was gradually increased at intervals of 1. For each fault number, 50 independently repeated experiments were randomly set up at the fault location, and two testing items for two kinds of body fluids of eight patients were completed using a modular and a routing-based fault repair method. The success rate of fault repair was obtained. The number of faulty electrodes was analyzed for each of the two methods. The relationship with the rate of failure repair was determined.

**Figure 8:**
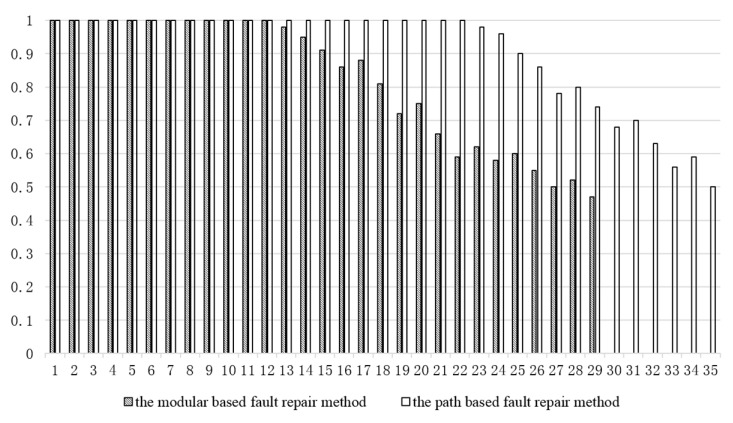
The number of faults and the repair rate of failure.

Figure 8 shows the bar statistics for the number of faults and the repair rate of failure as the number of faulty electrodes increased. The overall failure rate of repair was maintained at a total of 100% and then gradually declined. A failure repair rate of 100% could be guaranteed when the number of faulty electrodes was less than 12. 

At this time, the number of faulty electrodes was 12% of the total number of electrodes; when the number of faults was more than 28 (the faulty electrodes were 28% of the total number of electrodes), the fault repair rate was lower than 50%, and the chip has no means of repairing the fault. For the routing-based fault repair method, when the number of faulty electrodes was less than 23, a repair rate of 100% could be guaranteed. The number of faulty electrodes was 23% of the total number of electrodes. When the number of faults was more than 35 (35% of the total number of electrodes), the chip had no means of repairing the fault. From the experimental data, the routing-based fault repair method could guarantee a 100% fault repair rate for a large number of faulty electrodes and could improve the reliability and stability of the chip. When the number of faults was too large, it was necessary to sacrifice the completion time of the experiment to repair the fault of the chip, which led to a reduction in the fault repair rate.

**Figure 9:**
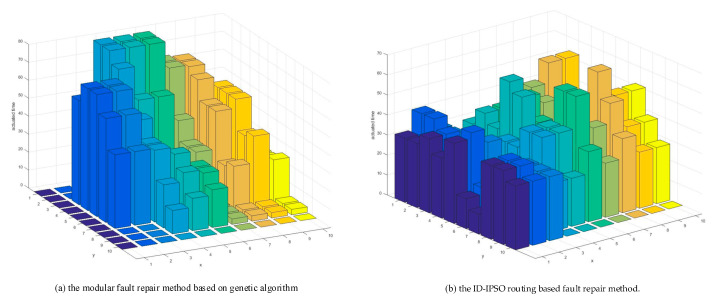
Comparison of the modular fault repair method based on the genetic algorithm and the heuristic routing-based fault repair method.

Five fault points were randomly set on the 10×10 electrode array, and two testing items for two body fluids of eight patients were completed using the modular method and the drop path method. This paper analyzed the use of electrodes based on modularization and path-based fault repair when chip failure occurred. Figure 9 presents a comparison of the modular fault repair method based on the genetic algorithm and the heuristic routing-based fault repair method. The number of electrodes used in a region based on the modular design method was higher, and the number of electrodes used in the path-based design method was more average.

Table 2 shows the usage of electrodes in modular and routing-based fault repair methods. Eighteen percent more electrodes were used in the routing-based fault repair method than in the modular method, and the electrode utilization rate was higher. The maximum electrode usage was 20.2% lower, and the average electrode usage was 17.0% lower. The more times an electrode was used, the more prone it was to failure. Therefore, comparing the two fault repair methods, the path-based method was safer.

## 5. Conclusions

The ID-IPSO routing fault repair method proposed in this study saved 14.2% of the experimental completion time on average compared to the greedy algorithm. The routing-based failure repair method averaged approximately 39.3% of the experimental completion time compared to the modular method. When the ratio of faulty electrodes was less than 12% and 23%, the modular and routing-based fault repair methods, respectively, could guarantee a 100% failure repair rate. The utilization rate of the electrodes was 18% higher than that of the modular method, and the average electrode usage time was 17%. Therefore, the ID-IPSO routing-based fault repair method could accommodate more faulty electrodes for the same fault repair rate; the experiment completion time was shorter, the average number of electrodes was lower, and the security performance was better. This study took the location of the fault point as assumed; however, in the process of the experiment, the electrodes on the chip may malfunction. In the future, the method of online fault repair should be studied on the basis of the design method proposed in this study, and the cleaning technology after the use of the chip should be further studied.

## Figures and Tables

**Table 1:** The design result of the health chip. ID-IPSO, improved Dijkstra and improved particle swarm optimization.

Size of DMFB	Completion Time of Experiment (s)	
	ID-IPSO Algorithm	Greedy Algorithm
8 × 9	59.22	68.77
8 × 8	59.21	69.13
7 × 8	59.36	69.46

**Table 2:** Comparison of the module-based and routing-based design methods.

	Use Rate of Electrode	Maximum Usage Times of Electrodes	Average Usage of Electrodes
Routing-based method	91%	59	39
Module-based method	73%	74	47
